# Cryptococcal osteomyelitis of the Zygomatic bone: a case report

**DOI:** 10.1186/s12879-020-05123-2

**Published:** 2020-06-05

**Authors:** Takashi Matsuki, Shunsuke Miyamoto, Taku Yamashita

**Affiliations:** Department of Otorhinolaryngology, Head and Neck Surgery, Kitasato University School of Medicine, 1-15-1 Kitasato, Minami-ku, Sagamihara, Kanagawa 252-0374 Japan

**Keywords:** Cryptococcosis, Osteomyelitis, Zygomatic bone, Fluconazole, Case report

## Abstract

**Background:**

Disseminated cryptococcosis is a well-characterized complication in immunocompromised patients with cryptococcal pneumonia or meningitis; however, isolated cryptococcal osteomyelitis is a rare entity that occurs in approximately 5% of patients with cryptococcosis. Cryptococcal osteomyelitis in the head and neck region is extremely rare. To the best of our knowledge, no cases of cryptococcal osteomyelitis affecting only the zygomatic bone have been reported to date.

**Case presentation:**

A 78-year-old man without other comorbidities presented with progressive swelling of the right cheek along with pain and trismus. Clinical examination revealed a tender swelling in the right zygomatic region; the maximal mandibular opening was about 2 cm. Laboratory data showed mildly elevated inflammatory indices (C-reactive protein: 0.45 mg/dL; erythrocyte sedimentation rate: 35 mm/h). Computed tomography showed a 30-mm-diameter lesion at the right zygomatic arch. A part of the lesion has extended to the subcutaneous area of the cheeks with signs of bone destruction and surrounding contrast effects. Histopathological examination of fine-needle aspirate and needle biopsy showed cryptococcus. Furthermore, culture of the aspirate showed growth of *Cryptococcus neoformans*. No evidence of any other site involvement was observed. Therefore, the patient was diagnosed with isolated cryptococcal osteomyelitis and was initiated on fluconazole therapy. The treatment was effective, and all symptoms were resolved in 4 weeks. Fluconazole therapy was stopped after 6 months. There are no signs of recurrence as of 15-month follow-up. The patient has no cosmetic abnormalities or sequelae.

**Conclusions:**

Fine-needle aspiration cytology, needle biopsy, and fungal culture were useful for definitive diagnosis. Immunocompetent patients with isolated osteomyelitis may be cured with oral fluconazole alone.

## Background

Cryptococcosis is an opportunistic infection that typically occurs in immunocompromised hosts such as patients with acquired immunodeficiency syndrome, hematologic malignancy, hepatic failure, and sarcoidosis, who underwent stem cell or solid organ transplant, or those on long-term steroid therapy. Disseminated cryptococcosis is a well-known complication of cryptococcal pneumonia or meningitis [1]. However, isolated cryptococcal osteomyelitis is a rare entity that accounts for approximately 5% of patients with cryptococcosis [2–4]. A systematic review revealed that only 89 cases of cryptococcosis are documented in the published literature [5, 6]. Cryptococcal osteomyelitis is usually caused by hematogenous spread from a primary lung infection after inhalation of microscopic airborne fungal spores [5, 6]. Direct inoculation of fungal spores due to trauma is another potential route of infection [5, 7]. Most reported cases of osseous cryptococcosis were due to *Cryptococcus neoformans* [3, 5]. Although the condition typically occurs in immunocompromised patients secondary to disseminated cryptococcosis, isolated osteomyelitis may occur in immunocompetent patients who have no apparent underlying disease or immune deficiency [1, 4, 8]. Systemic signs such as fever or fatigue are often not observed [3]. The most commonly reported sites of infection are the vertebrae, skull, and femur, respectively [5]. Infection of the zygomatic bone has been reported as a rare complication of otogenic infection such as otitis media or mastoiditis [9, 10]. Cryptococcal osteomyelitis in the head and neck region is extremely rare, and osteomyelitis of zygomatic bone alone has never been reported.

Here, we present the first documented case of cryptococcal isolated osteomyelitis of the zygomatic bone in an immunocompetent patient. Written informed consent of the patient has been obtained for publication of this case report and the accompanying images.

## Case presentation

**Fig. 1:**
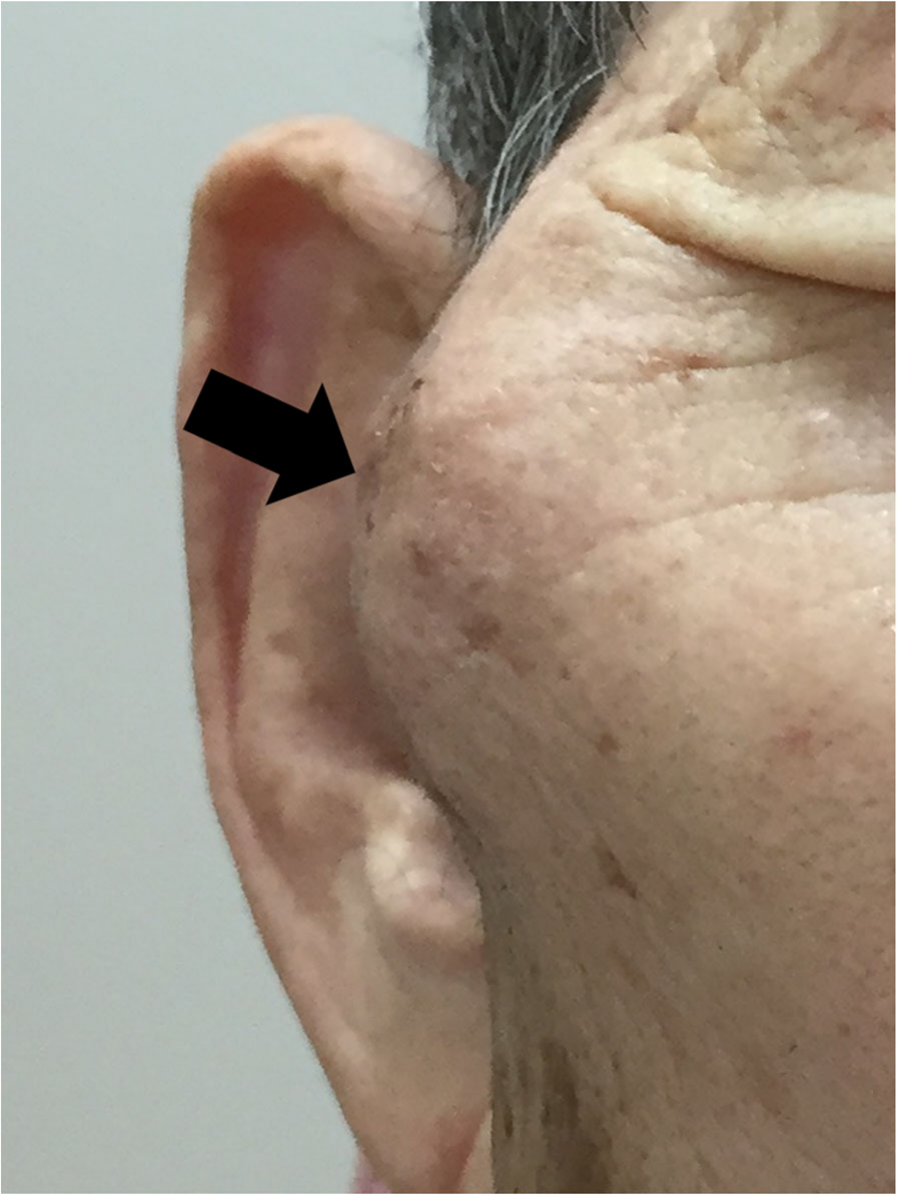
Photograph of the patient prior to initiation of treatment. The right zygomatic region shows swelling with erythema (arrow)

**Fig. 2:**
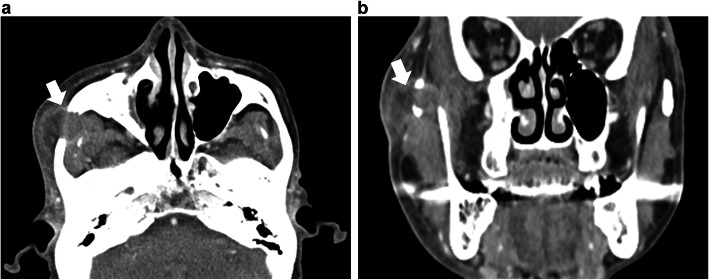
Pretreatment contrast-enhanced CT (**a**: axial **b**: coronal). The lesion (arrow) is located in the right zygomatic arch and has a maximum diameter of 30 mm; bone destruction and surrounding contrast effects are seen. A part of the lesion has extended to the subcutaneous area of the cheek

**Fig. 3:**
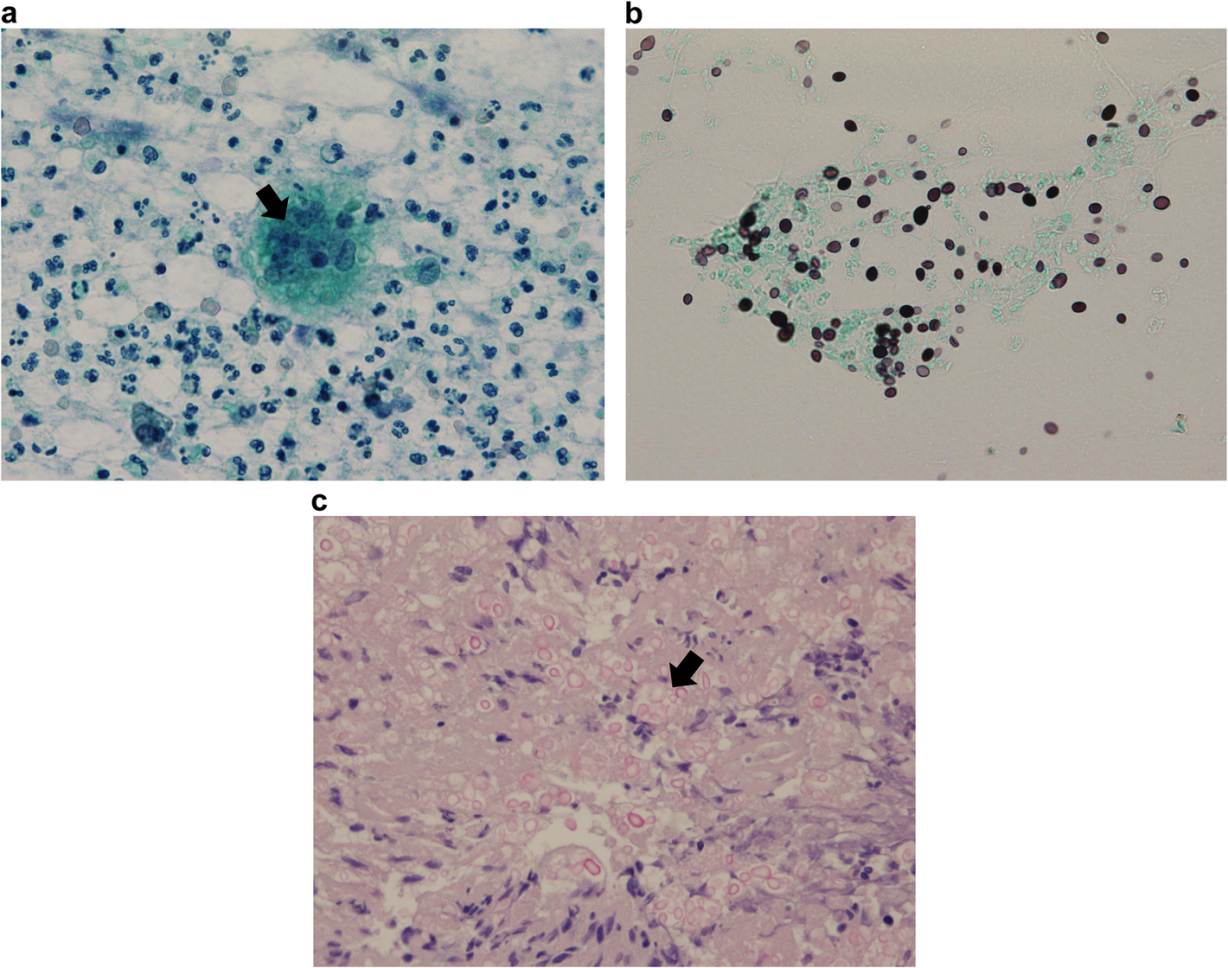
Histopathological findings. **a**: Papanicolaou stained smear showing several yeast-like cells phagocytosed by macrophage (arrow) (× 400). **b**: Grocott staining yielded positive results (× 400). **c**: Mucicarmine staining of biopsy specimen showing numerous cryptococci; yeast-like cells with stained mucopolysaccharide capsules are seen (arrow) (× 400)

**Fig. 4:**
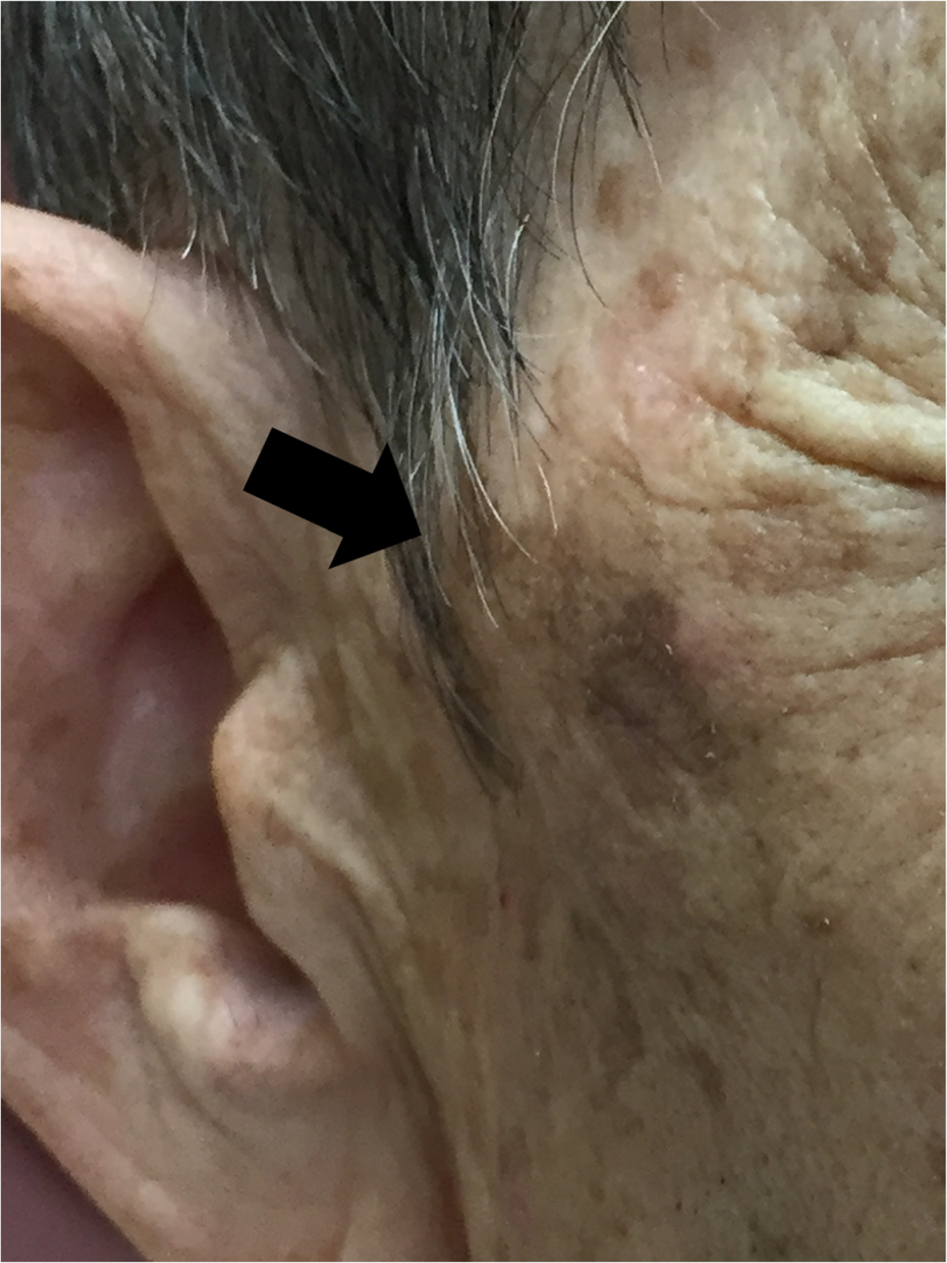
Photograph of the patient obtained 2 weeks after initiation of treatment. Swelling and erythema have resolved (arrow)

**Fig. 5:**
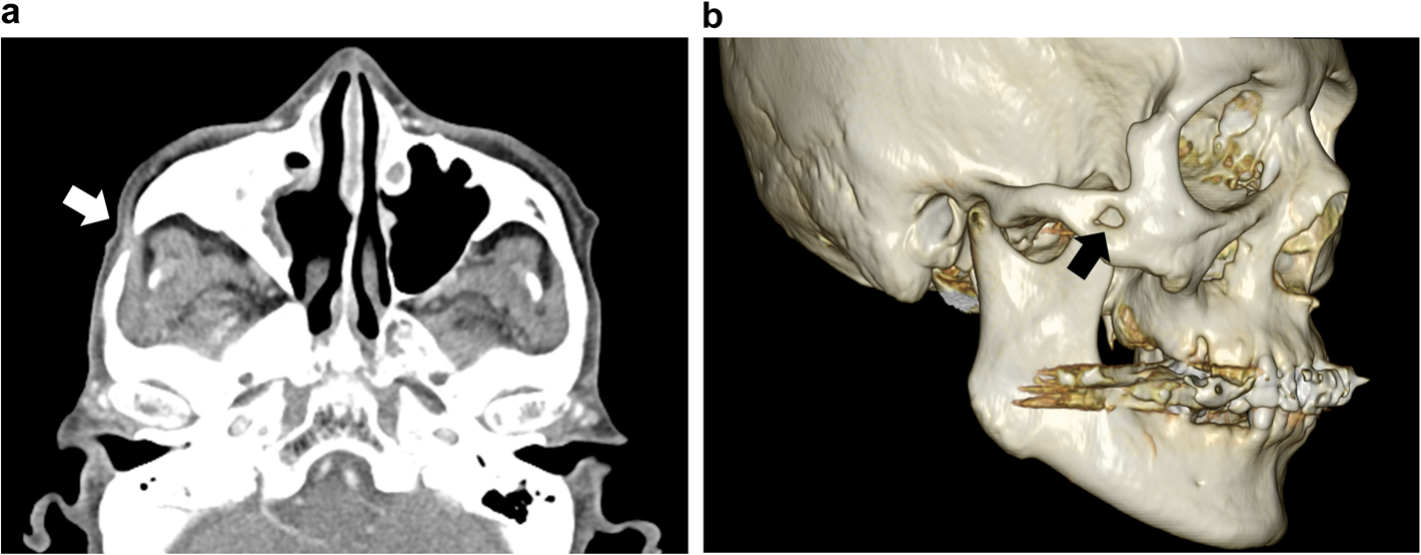
Contrast-enhanced CT (**a**: axial; **b**: three-dimensional imaging) 1 year and 3 months after completion of treatment. The lesion of the zygomatic region has resolved; a partial defect of the zygomatic bone is seen (arrow)

A 78-year-old man presented with a 2-week-long history of increasing right cheek swelling, pain, and trismus. He had no history of cheek trauma or fever. He had a history of prostate cancer that was treated with radiation therapy 2 years ago. He was not on any immunosuppressive treatment and did not experience previous recurrent or severe infections. He has no relevant exposures for *Cryptococcus*, such as that from birds or soil. There was no history of smoking or alcohol consumption. Clinical examination revealed swelling of the right zygomatic region (Fig. 1) with severe spontaneous pain and tenderness. His maximal mandibular opening was about 2 cm. On laboratory investigations, his serum C-reactive protein level was 0.45 mg/dL (reference range, 0–0.14), total white blood cell count was 6700/mm^3^ (reference range, 3300–8600) with 54.4% neutrophils, and erythrocyte sedimentation rate (ESR) was 35 mm/h (reference range, 2–10). Serum levels of alkaline phosphatase, calcium, phosphorous, glucose, and glycosylated hemoglobin A1c were within the normal range. His serum alanine aminotransferase was 11 IU/L (reference range, 10–42) and aspartate transaminase level was 22 IU/L (reference range, 13–30). The viral serology tests for hepatitis B and hepatitis C yielded negative results. Contrast-enhanced computed tomography (CT) scan showed the lesion in the right zygomatic arch with a maximum diameter of 30 mm. There were signs of bone destruction and surrounding contrast effects (Fig. 2). The lesion had extended to the subcutaneous tissue in the cheek; however, there were no signs of hardening or calcification. These findings are suggestive of a malignant tumor derived from the zygomatic bone or a focal abscess with features not typical of an infection caused by common mechanisms such as by extension of an otogenic infection. *Cryptococcus* was found in the fine-needle aspiration cytology (FNAC) based on Grocott staining; additional histopathological diagnosis of needle biopsy from the mass was non-necrotizing granuloma with *Cryptococcus* (Fig. 3). Furthermore, culture of the aspirate showed growth of *Cryptococcus neoformans*, and the minimal inhibitory concentration of fluconazole for cryptococcus was low at 2.0 μg/ml. Tests for human immunodeficiency virus (HIV) antigens and antibodies were negative. Lumbar puncture (LP) revealed clear and colorless cerebrospinal fluid (CSF) with an opening pressure of 70 mmH_2_O. The CSF cell count was < 1/μL (reference range, 0–5), and glucose and protein levels were within normal limits [67 mg/dL (reference range, 50–75) and 34 mg/dL (reference range, 10–40), respectively]. The CSF tested negative for cryptococcal antigens. CSF and blood cultures showed no bacterial or fungal growth. Brain magnetic resonance imaging (MRI), and chest CT. Finally, the patient was diagnosed with isolated cryptococcal osteomyelitis of the zygomatic bone and initiated on fluconazole therapy (400 mg daily). By 2 weeks, the swelling remarkably improved with alleviation of both trismus and pain. By 4 weeks, the swelling had disappeared (Fig. 4). Fluconazole therapy was stopped after 6 months. Follow-up examination at 15 months showed a very small palpable depression in the zygomatic area; however, there were no signs of lesion recurrence (Fig. 5). Inflammatory markers including ESR and CRP remained negative. There were no cosmetic abnormalities or sequelae.

## Discussion and conclusions

Isolated cryptococcal osteomyelitis is a rare entity [2–4]. According to previous reports, including systematic reviews [5, 11], the predominant complaints included soft tissue swelling and pain [5]. On the laboratory tests, ESR which reflects bone infection was elevated frequently [5]. Both FNAC and open biopsy were commonly performed and found to be useful for the diagnosis. However, in recent years, FNAC is recommended owing to its minimally invasive nature [5]. Fungal cultures have been reported to show a high positive rate and *Cryptococcus neoformans* was identified the most frequently as the causative organism [5]. Imaging findings of cryptococcal osteomyelitis have no typical features, and several previous case reports have documented lesions mimicking malignant tumors [5, 11]. The treatment strategy greatly depends on the absence or presence of disseminated infection. To rule this out, several examinations such as brain MRI, chest CT, LP, tests of serum and CFS cryptococcal antigen titer, and fungal blood and CSF cultures were performed. In addition, a past history, comorbidities such as diabetes, and serology tests for hepatitis virus and HIV are referenced to identify immunodeficiency. Except for the infection of lungs and central nervous system, there are no standardized treatment protocols for cryptococcal infection of specific body sites. A combination of antifungal therapy and surgical debridement has been used to treat many patients with osseous cryptococcosis [3, 5, 12, 13]. According to the Infectious Disease Society of America, oral fluconazole (400 mg per day for 6–12 months) is the treatment of choice for immunocompetent patients with non-meningeal, non-pulmonary cryptococcosis [5, 14, 15]. Several case reports have documented successful treatment of patients with isolated cryptococcal osteomyelitis with fluconazole alone due to good oral availability [3, 5, 14, 16]. Although the outcomes of disseminated cryptococcosis are typically unfavorable, immunocompetent patients with isolated osteomyelitis have a good prognosis [5, 17].

Our patient presented with typical chief complaints and laboratory data of mild elevation of ESR. Owing to the localized inflammation, no systemic signs such as fever or fatigue were observed. We performed both FNAC and a needle biopsy to obtain a definitive diagnosis of this very rare disease. However, as FNAC clearly showed cryptococcal infection in our patient, an invasive needle biopsy may not have been necessary. The culture of the aspirate showed the growth of *Cryptococcus neoformans,* the most major causative organism*.*

We couldn’t diagnose only by the imaging findings. The main differential diagnosis of trauma, extension of otogenic bacterial infection, and malignant bone tumor were excluded based on the absence of trauma history, CT findings and laboratory data, and histopathological diagnosis, respectively. By the results of several examinations that performed generally, we ruled out disseminated infection and diagnosed our case as isolated osteomyelitis in an immunocompetent patient consequently. However, the serum cryptococcal antigen levels should have been examined, and thereby, additional tests should be performed for cellular and innate immunity, such as that for CD4 lymphopenia, lymphocyte subsets, and serum immunoglobulins, to rule out immunodeficiency caused by factors other than aging.

Since the excision of the zygomatic bone would have caused cosmetic defects, our patient was treated with oral fluconazole alone and was successfully cured. If cryptococcal resistance to fluconazole was observed, another effective antifungal agent would have been administered instead of the surgical treatment. Our patient showed no clinical and radiographical signs of recurrence; however, a palpable partial defect of the zygomatic bone remained. Moreover, there was no gross discrepancy between the two sides of the face on visual examination. The zygomatic bone is a key element of the craniofacial skeleton and plays an important role in maintaining the face shape [18]. Previous reports suggested that it is desirable to reconstruct surgical defects or deformation of the zygomatic bone caused by fracture [19, 20]. In our patient, the shape of the zygomatic bone was maintained after treatment. However, the presence of a large defect or occurrence of secondary fracture may have caused cosmetic deformities as well as ocular or mandibular dysfunction. In addition, because the masticatory muscles and the temporomandibular joint are in close proximity to this bone, trismus is liable to develop due to the spread of inflammation, as seen in our patient. In case of prolonged and intense inflammation, trismus may not completely resolve after treatment. For these reasons, early diagnosis and treatment of zygomatic lesions are desirable.

Our case may have a risk of secondary fracture or recurrence in the future. For secondary fractures with dysfunction, fixation surgery will be performed. In addition, fluconazole can be re-administered, which has been found effective for recurrence.

In summary, we report the first case of cryptococcal isolated osteomyelitis of the zygomatic bone in an immunocompetent patient without history of trauma. Although the imaging findings were atypical, FNAC, needle biopsy, and fungal culture were useful in obtaining a definitive diagnosis. Patients with isolated osteomyelitis and may be cured with oral fluconazole alone.

## Data Availability

The main data used or analyzed in this case report are included in this published article. More detailed data are available from the corresponding author on reasonable request.

## Abbreviations

ESR: Erythrocyte sedimentation rate
CT: Computed tomography
FNAC: Fine-needle aspiration cytology
HIV: Human immunodeficiency virus
LP: Lumbar puncture
CSF: Cerebrospinal fluid
MRI: Magnetic resonance imaging
